# Combined Application of Cold Physical Plasma and Chemotherapeutics against Chondrosarcoma Cells

**DOI:** 10.3390/ijms25136955

**Published:** 2024-06-25

**Authors:** Andreas Nitsch, Sara Qarqash, Frank Schulze, Lars Nonnenmacher, Sander Bekeschus, Mladen V. Tzvetkov, Georgi I. Wassilew, Lyubomir Haralambiev

**Affiliations:** 1 Center for Orthopedics, Trauma Surgery and Rehabilitation Medicine, University Medicine Greifswald, Ferdinand-Sauerbruch-Str., 17475 Greifswald, Germany; 2 ZIK Plasmatis, Leibniz Institute for Plasma Science and Technology (INP), Felix-Hausdorff-Straße 2, 17489 Greifswald, Germany; 3 Clinic and Policlinic for Dermatology and Venerology, Rostock University Medical Center, Strempelstr. 13, 18057 Rostock, Germany; 4 Department of General Pharmacology, Institute of Pharmacology, Center of Drug Absorption and Transport (C_DAT), University Medicine Greifswald, 17487 Greifswald, Germany

**Keywords:** cancer, CAP, cisplatin, cold atmospheric plasma, CPP, doxorubicin, medical plasma, NIPP, vincristine

## Abstract

Chondrosarcoma (CS) is a rare malignant bone sarcoma that primarily affects cartilage cells in the femur and pelvis. While most subtypes exhibit slow growth with a very good prognosis, some aggressive subtypes have a poorer overall survival. CS is known for its resistance to chemotherapy and radiotherapy, leaving surgery as the sole effective therapeutic option. Cold physical plasma (CPP) has been explored in vitro as a potential therapy, demonstrating positive anti-tumor effects on CS cells. This study investigated the synergistic effects of combining CPP with cytostatics on CS cells. The chemotherapeutic agents cisplatin, doxorubicin, and vincristine were applied to two CS cell lines (CAL-78 and SW1353). After determining their IC_20_ and IC_50_, they were combined with CPP in both cell lines to assess their impact on the cell proliferation, viability, metabolism, and apoptosis. This combined approach significantly reduced the cell proliferation and viability while increasing the apoptosis signals compared to cytostatic therapy alone. The combination of CPP and chemotherapeutic drugs shows promise in targeting chemoresistant CS cells, potentially improving the prognosis for patients in clinical settings.

## 1. Introduction

Chondrosarcomas (CSs) are a diverse group of tumors characterized by the slow formation of the cartilage matrix. Accounting for 20% of malignant bone tumors, CS is the second most common type of bone cancer, right after osteosarcoma [1]. The worldwide incidence is approximately 1.2–3.4 cases per million per year, varying among different populations [2]. CSs typically manifest in older individuals and have a slight male preponderance, with the peak incidence in the sixth and seventh decades of life [3,4]. They are primarily located in the femur, pelvis, humerus, and ribs [5].

In 2020, the World Health Organization (WHO) reclassified bone tumors, considering the histology, molecular characteristics, and biological behavior, resulting in the categorization of CSs into eight different subtypes [6] with regard to their prognosis. While atypical cartilaginous tumors (ACT) and secondary chondrosarcomas have a great 5-year survival rate of ca. 90%, the survival declines with increasing tumor grades, reaching 30% for grade III chondrosarcomas [7]. Additionally, the prognosis for high-grade CS seems to worsen when patients develop distant metastasis [8,9]. Another important prognosis factor is the development of a local recurrence (LR) after surgery [10]. Stevenson J. et al. [11] showed in their study that the surgical margins have a significant impact on LR in all the CS subtypes. This poses a challenge in the treatment of large tumors and challenging cases of CS, such as those located in the pelvis, as adequate surgical resection remains the primary therapeutic option [4,12].

One of the most important weapons against bone tumors is the application of chemotherapy. Since the introduction of chemotherapeutic agents in oncology, the survival rate of patients has improved tremendously. Chemotherapeutic agents such as cisplatin (CIS), doxorubicin (DOX), and vincristine (VIN) are part of the standard sarcoma treatment protocol. While being effective in the treatment of many different malignancies, traditional chemotherapeutic agents and radiotherapy have unfortunately proven ineffective in the treatment of CSs [13,14]. Their general insensitivity to chemotherapy may be due to several factors, including the high expression of antiapoptotic protein Bcl-2 and the presence of P-glycoprotein [15,16]. P-glycoprotein is a marker of the presence of drug resistance against several chemotherapeutic agents, including those investigated in this study: CIS, DOX, and VIN [15].

Cold physical plasma (CPP), a non-thermal ionized gas primarily composed of reactive oxygen species (ROS) and reactive nitrogen species (RNS), has emerged as a promising therapeutic approach for cancer [17,18]. Investigations in vitro, in vivo, and in clinical studies have consistently shown CPP’s anti-tumor effects when applied to cells from various cancer types [19,20,21,22,23,24]. In CS, CPP has shown similar effects, including the inhibition of cell growth and viability, as well as the induction of apoptosis [25,26]. Even though the mechanisms are not yet fully understood, new studies suggest that CPP could enhance the cell sensitivity to chemotherapeutic agents and, in some cases, help to overcome drug resistance [27,28,29,30].

This study aims to explore the effects of CPP in combination with chemotherapeutic agents on CS cells in vitro, with the goal of enhancing the overall efficacy of chemotherapy in the treatment of CS. The proposed approach has the potential to reduce the LR and thereby improve the overall patient survival.

## 2. Results

**Figure 1:**
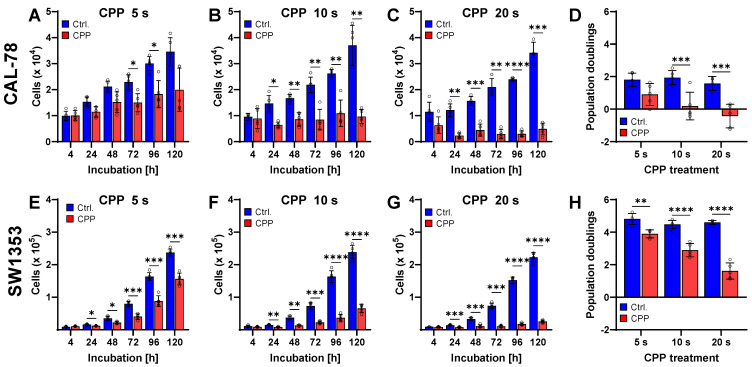
CPP treatment reduces cell proliferation in chondrosarcoma (CS) cell lines CAL-78 (**A**–**D**) and SW1353 (**E**–**H**). The human CS cell lines were treated with cold physical plasma (CPP), and the control treatment was the carrier gas argon (Ctrl.). The viable cells were counted at the indicated times using the CASY Cell Counter and Analyzer (**A**–**C**,**E**–**G**). The population doublings were calculated (**D**,**H**). Data are presented as individual values and mean ± SD. The differences between CPP and control were tested for significance using a two-way repeated measure ANOVA followed by Bonferroni’s multiple comparison tests (**A**–**C**,**E**–**H**) or a two-way ANOVA followed by Bonferroni’s multiple comparison tests (* *p* < 0.05, ** *p* ≤ 0.01, *** *p* ≤ 0.001, **** *p* ≤ 0.0001). Results from five independent experiments.

In order to investigate the influences of CPP on CS, the human CS cell lines CAL-78 (Leibniz Institute DSMZ-German Collection of Microorganisms and Cell Cultures, Braunschweig, Germany) and SW1353 (American Type Culture Collection, Manassas, VA, USA) were exposed to CPP. A single CPP application leads to a reduction in the cell proliferation of CS cells. To examine the influences of CPP on CS cells, they were treated with CPP for 5 s, 10 s, and 20 s. In both CS cell lines employed, CPP led to a significant reduction in cell proliferation (*p* < 0.0001). The effects depended on the treatment time and ranged from a reduction in the cell number after 120 h by approximately 50% (CAL-78) or 30% (SW1353) to a complete inhibition of growth (Figure 1). The connection between the treatment duration and the decrease in cell proliferation becomes particularly clear when looking at the population doublings (Figure 1D,H). Here, even negative values were achieved after a 20 s CPP treatment of the CAL-78 cell line, indicating that cell death exceeded cell growth. While comparing the two CS cell lines employed, the CAL-78 cells reacted more sensitively to the CPP treatment than the SW1353 cells.

**Figure 2:**
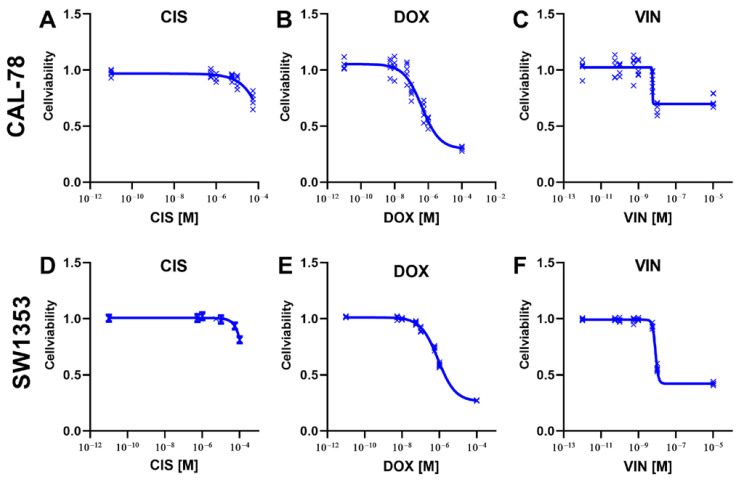
The cell viability of human chondrosarcoma (CS) cells was measured after treatment with the cytostatic drugs cisplatin (CIS) (**A**,**D**), doxorubicin (DOX) (**B**,**E**), and vincristine (VIN) (**C**,**F**). The cells were treated with the indicated drugs and incubated for 72 h. The cell viabilities were determined using the CellTiter-Blue reagent. The measured fluorescence signals of the cytostatic-treated cells were normalized to the signal of the DMSO-treated control cells. Results from five independent experiments.

Dose–response curves were created to determine the efficacy of the different chemotherapeutic agents in relation to CS cells. DOX was the most effective agent, reaching an inhibition of more than 50% in both cell lines. With the cytostatic drug VIN, this strong effect was observed in the cell line SW1353. Treatment with a cytostatic drug alone did not achieve a more than 50% inhibition in any of the other examined combinations of cytostatic agent and cell line. CIS was extremely inefficient for both target lines (Figure 2).

**Figure 3:**
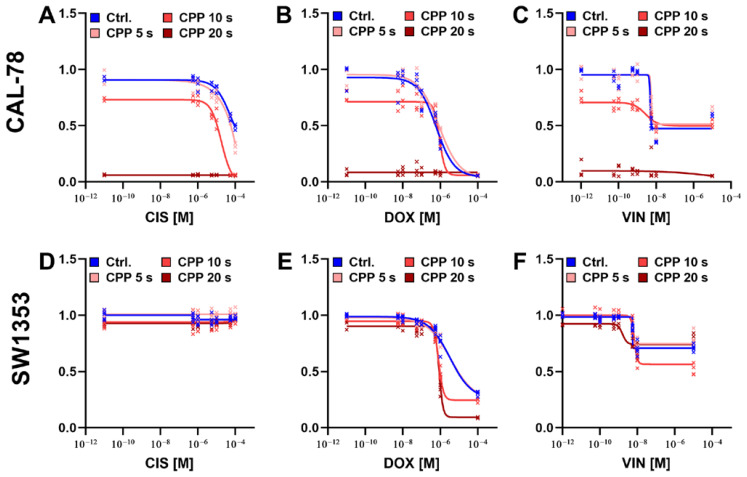
Combined treatment of human chondrosarcoma (CS) cells with cytostatic drugs and cold physical plasma (CPP). CAL-78 (**A**–**C**) and SW1353 (**D**–**F**) CS cell lines were treated with cisplatin (CIS), doxorubicin (DOX), and vincristine (VIN). The cells were also treated with CPP for 5 s, 10 s, and 20 s. The cell treated with cytostatics and carrier gas argon served as a control. The cell viabilities were determined with CellTiter-Blue. The measured fluorescence signals of the cytostatic-treated cells were normalized to the signal of the DMSO-treated control cells. Results from at least three independent experiments.

The effect of the combined treatment of CPP and the different chemotherapeutic agents was investigated to establish dose–response curves for the respective combinations and treatment times. For the CAL-78 cells, an synergistic effect of the combination of CPP and the cytostatic drugs was demonstrated. The observed effect was strongly dependent on the treatment duration with CPP. With an increasing treatment time, the cytostatic drug concentration required for a comparable inhibitory effect was found to decrease. Overall, the effects on the SW1353 cell line were less pronounced. However, the treatment combination of CPP and DOX was found to be the most effective. The inhibitory effect of VIN on the SW1353 cells was also observed to be increased (Figure 3).

**Figure 4:**
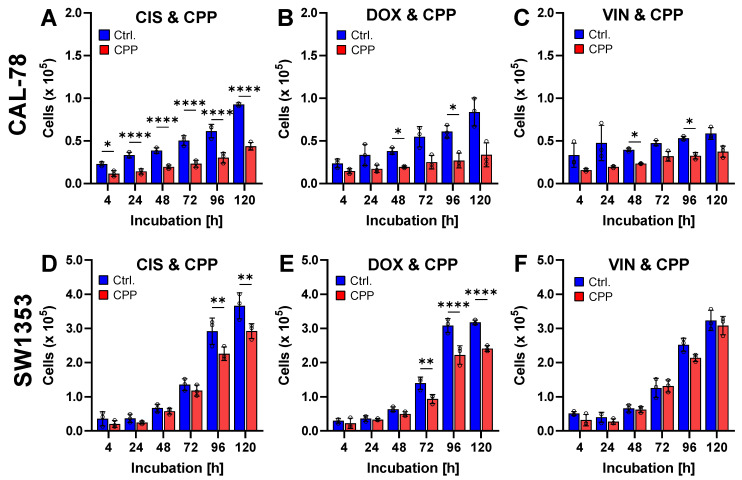
The effect of combination therapy with cold physical plasma (CPP) and cytostatic drugs of chondrosarcoma (CS) cells. Human CS cells from CAL-78 (**A**–**C**) and SW1353 (**D**–**F**) were treated with cisplatin (CIS), doxorubicin (DOX), and vincristine (VIN) in combination with CPP (red) or carrier gas argon (blue). The cells were incubated for 120 h. The viable cells were counted at the indicated time points using the Invitrogen Countess II Cell Counter. The graphs show the individual cell numbers and mean values ± SD and a two-way-repeated measure ANOVA was used (* *p* < 0.05, ** *p* ≤ 0.01, **** *p* ≤ 0.0001). Results from three independent experiments.

The effect of the combination of different cytostatic agents and CPP on the cellular viability was tested 120 h after exposure (Figure 4). In this context, the combination of DOX and CPP has been proven to be particularly effective. In the CAL-78 cells, a significant reduction in cell proliferation was achieved through the combination of chemotherapeutics and CPP. Similarly, in the SW1353 cells, an additional treatment with CPP also significantly inhibited proliferation.

**Figure 5:**
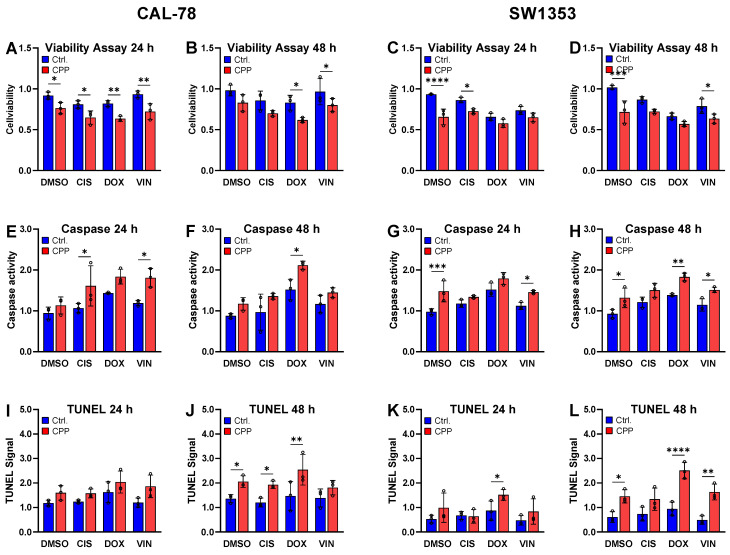
Cytotoxicity assay, detection of apoptosis using caspase 3/7 activity assay and TUNEL assay. The human chondrosarcoma (CS) cell lines CAL-78 (**A**,**B**,**E**,**F**,**I**,**J**) and SW1353 (**C**,**D**,**G**,**H**,**K**,**L**) were treated with isolated DMSO, cisplatin (CIS), doxorubicin (DOX) and vincristine (VIN) or a combination of the drug and CPP. Cytotoxicity assay (**A**–**D**), caspase 3/7 activity assay (**E**–**H**) and TUNEL assay (**I**–**L**) were used to determine apoptosis. Individual values and means ± SD are shown in the graphs and were tested for statistically significant differences using a two-way ANOVA followed by Bonferroni’s multiple comparison tests (* *p* < 0.05, ** *p* ≤ 0.01, *** *p* < 0.001, **** *p* ≤ 0.0001). Results from three independent experiments.

The cell viability assays demonstrated that the cell viability was significantly reduced as early as 24 h after treatment (Figure 5A–D). Treatment with the chemotherapeutics alone, at the previously determined IC_20_, resulted in a significant reduction in the cell viability. Moreover, the cytotoxic effect was further enhanced by combining the chemotherapeutics with CPP. The apoptosis induction assays (Figure 5E–L) corroborated these findings. The chemotherapeutics alone induced apoptosis, and this effect was further amplified by additional treatment with CPP.

**Figure 6:**
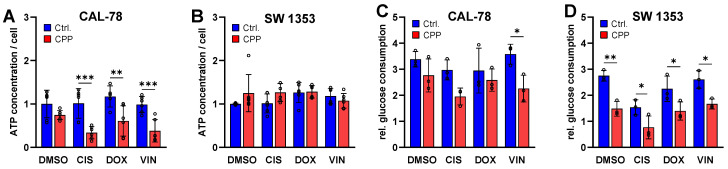
Detection of the relative ATP concentration and the glucose consumption. Human chondrosarcoma (CS) cell lines CAL78 (**A**,**C**) and SW1353 (**B**,**D**) were treated with isolated DMSO, cisplatin (CIS), doxorubicin (DOX), and vincristine (VIN) or a combination of the drug and 5 s cold physical plasma (CPP). The relative ATP concentration was determined with CellTiter-Glo and normalized to the cell viability determined with the CyQUANT assay. The glucose consumption of the cells was analyzed with the Glucose-Glo assay. The mean values ± SD are depicted in the graphs and were tested for statistically significant differences using a two-way ANOVA followed by Bonferroni’s multiple comparison tests (* *p* ≤ 0.05, ** *p* ≤ 0.01, *** *p* ≤ 0.001). Results from five (**A**,**B**) or three (**C**,**D**) independent experiments.

To investigate the influence of CPP in combination with different chemotherapeutic agents on the cell metabolism, the cellular ATP concentration within the cells and the glucose consumption of the cells were determined (Figure 6). It was shown that treatment with cytostatic alone did not lead to a significant reduction in the ATP concentrations. The experiments on glucose consumption showed similar results. Here, only treatment with cisplatin led to a reduction in the glucose consumption. Additional CPP treatment resulted in significantly reduced cell metabolism. However, the effects here were cell line-specific. While a reduction in the intracellular ATP concentration was found in the CAL-78 cells, this effect was hardly detectable in the SW1353 cells. There was a significant reduction in the glucose consumption in both cell lines as a result of the CPP treatment, although the effects were more pronounced in the SW1353 cells.

In particular, the treatment of the CAL-78 cells led to a reduction in ATP. The combination of the cytostatic agent and CPP proved to be particularly effective. As observed for the apoptosis rate, the effects were cell line-specific and more pronounced in the CAL-78 cells.

**Figure 7:**
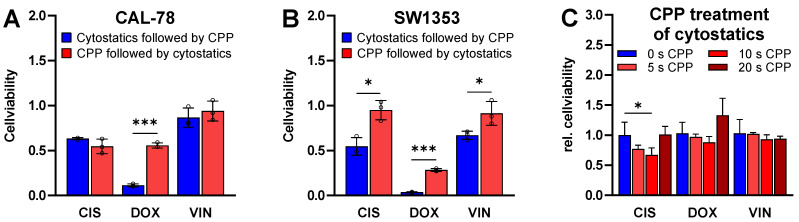
Sequential treatment and the influence of CPP on chemotherapeutics. CAL-78 (**A**) and SW1353 (**B**) cells were treated in two different orders: first with chemotherapeutics followed by CPP, and vice versa. The cell viability was detected with CellTiter-Blue. The cytostatics were pre-treated with CPP for 0 s, 5 s, 10 s and 20 s. After 48 h, the SW1353 cells were treated with the CPP-pre-treated cytostatics and the cell viability was measured after 48 h incubation with CellTiter-Glo (**C**). The mean values ± SD are depicted in the graphs and were tested for statistically significant differences using a two-way ANOVA followed by Bonferroni’s multiple comparison tests (* *p* ≤ 0.05, *** *p* ≤ 0.001). Results from three independent experiments.

To determine the impact of sequential treatment, the cells were treated in two different orders: first with chemotherapeutics followed by CPP, and vice versa (Figure 7A,B). It was found that treating the cells with CPP after chemotherapeutic pre-treatment was more effective than the reverse. These effects were observed in both cell lines and with all the investigated chemotherapeutics. However, the effects were particularly pronounced in the treatments with DOX.

To investigate a potentially direct impact on the efficacy of cytostatics, CIS, DOX and VIN were treated with CPP. The results indicated no critical impact of the CPP treatment on the efficacy of the cytostatics (Figure 7C). There was a slight trend suggesting that pre-treatment of the cytostatics with up to 10 s of CPP might lead to a further reduction in the cell viability. However, only the combination of DOX and 20 s of CPP pre-treatment showed a minimal trend toward the reduced efficacy of DOX due to pre-treatment with CPP.

## 3. Discussion

Since its potential anti-tumor effect was first described in 2007 [31], CPP has been recognized as a promising approach for the treatment of various types of cancers [32,33,34,35,36,37]. It exerts its effects by increasing the cellular level of reactive and nitrogen species (RONS), triggering DNA double-strand breaks, cell cycle arrest, and apoptosis [38]. Furthermore, CPP is believed to enhance the efficacy of chemotherapeutic agents when used in combination. Recent studies have revealed a potentially synergistic effect with the combined use of CPP and chemotherapy in different cancer types, like glioblastoma [39,40,41], melanoma [29,42,43], breast cancer [44,45,46], pancreatic adenocarcinoma [47,48] and Ewing sarcoma [49].

In our prior studies on CS, we observed that CPP can inhibit cell growth, induce programmed cell death, and diminish cell migration [25,26]. The current study reaffirms this observation, confirming the inhibition of cell growth upon CPP treatment. This aligns with findings from other studies, where a linear increase in the growth inhibition correlates with the duration of exposure to CPP [50,51]. CS is known to be resistant to chemotherapy and radiotherapy, leaving surgeons with resection as the main treatment option [52]. The resistance to chemotherapy in CS is linked to the slow doubling time of its cells and the expression of the multidrug-resistance-1 gene, particularly the P-glycoprotein [15,53]. Some studies suggest that certain subtypes of CS may respond to combined chemotherapy, but no improvement in the overall patient survival rate has been achieved yet [54].

Given the positive effects of CPP and the limited success of conventional treatments, the current study explores the potential synergy of combining CPP with CIS, DOX, and VIN for the treatment of CS. In their study, Van Oosterwijk et al. [55] demonstrated that various CS cell lines showed the best response to DOX and limited or no efficacy with CIS, indicating varied sensitivities among different cell lines. This aligns with our observations, where DOX achieved the best inhibition of over 50% in both cell lines, SW1353 and CAL-78, while CIS showed inefficiency in both target lines. The effects of treating CS with VIN alone have not yet been studied. Some studies have explored combining VIN with other chemotherapeutics for the treatment of soft tissue sarcoma, revealing the limited effectiveness of these regimens on such sarcomas [56].

Combining CPP with chemotherapy on CAL-78 cells significantly decreased the cell viability compared to the control treatment with argon gas and chemotherapy. The impact became more pronounced with longer CPP treatment. While the 5-s CPP application was less effective in reducing the IC_20_ and IC_50_ of all the cytostatics, the 20-s application nearly wiped out the CAL-78 cell viability. Interestingly, the SW1353 cells maintained resistance to CIS even after the combined treatment with CPP. In addition, these cells exhibited less sensitivity to the combination treatment with VIN compared to the CAL-78 cells. DOX continued to be the most effective chemotherapeutic agent in the treatment of the SW1353 cell line in both sole and combined therapy.

Furthermore, the effect of the combination therapy with CPP and cytostatic drugs on the cancer cell metabolism was investigated by measuring the cellular ATP concentration and glucose consumption as an indicator of metabolically active cells. Malignant cells frequently utilize anaerobic respiration to fulfill their metabolic requirements [57]. During this process, glucose is converted into pyruvate, resulting in the production of ATP and lactate. The export of lactate to the tumor microenvironment is known to promote cancer growth through multiple mechanisms [58]. Consequently, the impact of the combination of CPP and cytostatics on the glucose metabolism of CS represents an intriguing indicator [59]. To investigate the influence of combined CPP and cytostatic therapy on the metabolism of CS cells, two different methods were employed in the current study. On the one hand, the cellular ATP concentration in the cells was quantified, and on the other hand, the glucose consumption of the malignant cells was determined. Treatment with cytostatic drugs alone did not result in a reduction in the ATP levels or changes in the glucose consumption of the CS cells. Only the additional CPP application, i.e., the combined treatment, led to a significantly reduced cell metabolism. Here, too, the extent of the effects was cell line-specific. While the combined therapy caused a greater reduction in the intracellular ATP concentration in the CAL-78 cells than in the SW1353, this combined application was more pronounced in the SW1353 cell line in terms of the glucose consumption. The analysis of the results from numerous studies investigating the effects of CPP on malignant cells indicates that different cell lines of the same malignant entity do not respond equally to CPP [25,34,60].

After combining CPP with the respective chemotherapeutics, there was not a significant increase in the growth inhibition of the CAL-78 and SW1353 cells. Since the single treatments alone already completely halted the cell growth, an additional effect due to the combination with CPP was not possible.

An important point regarding the combination of CPP with cytostatics is the possibility of interaction between those two methods. It was demonstrated that CPP pre-treatment of chemotherapeutic agents does not compromise their efficacy. In our experiment, we treated the cytostatics with CPP and then incubated them for 48 h. After the CPP application on cytostatic, an incubation period of 48 h was chosen to ensure that the CPP-generated reactive species were no longer active when the medium containing the CPP-treated cytostatics was applied to the cells. The activity of those that are reactive normally lasts for very short durations [61,62]. These findings provide evidence of the efficacy of combining CPP with chemotherapeutic agents. The cytostatics themselves appear to be resilient against the effects of CPP treatment, as demonstrated by the results indicating that their effectiveness remains largely unaltered by CPP pre-treatment in our experimental setup. Furthermore, other studies employing a combination of CPP with cytostatics, such as doxorubicin, corroborate the current findings, indicating that CPP application does not impact the efficacy of the cytostatic drugs [28,44,49].

One of the key anticancer effects induced by CPP is programmed cell death, specifically through apoptosis [63,64], which has been well-documented in various cancer types [65,66,67]. The current study confirmed that a single 5-s CPP treatment of CAL-78 cells resulted in significant apoptotic effects, evidenced by an increase in the caspase 3/7 activity and TUNEL signals at 24 and 48 h after exposure. However, these effects were not observed when treating the SW1353 cells with CPP. In a previous study [25], it was demonstrated that CPP treatment increased the apoptotic signals in both SW1353 and CAL-78 cells. However, compared to the 5 s CPP treatment used in this study, the treatment duration in that work was doubled to 10 s. This suggests a potential correlation between the duration of CPP exposure and the activation of apoptotic pathways in CS cells. The combination treatment with CPP and various chemotherapeutic agents resulted in a slight increase in the activation of apoptotic mechanisms in the CAL-78 cell line. The effects were evident through the increased caspase 3/7 activity but not the TUNEL signals. These effects were more pronounced 48 h after treatment. However, in the SW1353 cells, the combination treatment did not lead to an increase in the apoptotic signals. This could be attributed to the short duration of the CPP application, which was only 5 s. In a study on breast cancer, Zahedian S. et al. [45] showed an increase in the apoptotic cell percentages after combination therapy with CPP and DOX compared to DOX alone. Lee CM et al. [68] demonstrated higher apoptotic signals in oral squamous cancer cells when treated with CPP and CIS compared to CIS alone. Yet, it is important to highlight that both studies applied CPP for longer durations of 1 and 3 min, which are significantly longer than the treatment time employed by us. This emphasizes the need to explore the apoptotic effects of the combination therapy of cytostatics and CPP for CS with a longer CPP application time.

This study suggests that the combined use of CPP and cytostatics enhances the effects of chemotherapeutic agents, potentially increasing the sensitivity of CS cells to these treatments. Additionally, this combination may lead to a decrease in the effective dose of the applied cytostatic while achieving a more potent cytotoxic effect on CS cells. This approach not only minimizes the side effects of cytostatics but also holds promise in improving the overall treatment outcomes and subsequent patient survival. The application of CPP could take place during the surgical resection of the tumor, either through direct application or by rinsing with CPP-activated liquids as an indirect CPP treatment, targeting the tumor margins. This approach, in addition to adjuvant chemotherapy, could be particularly beneficial for large tumors or challenging chondrosarcoma cases with elevated rates of positive margins and the associated complications.

This study, for the first time, shows the effects of combined therapy involving CPP and chemotherapeutic agents on chondrosarcoma cells, offering insights into new treatment options for this condition and indicating that the known resistance to chemotherapeutic agents can be overcome.

Several safety studies have been performed, showing no significant side effects of the application of CPP in vivo [69,70,71]. The results of patient studies also indicate that the use of CPP in the oncological field is safe [72,73].

One limitation of this study is that it was conducted in a cell culture model. Further studies, including animal experiments, are necessary to confirm these findings in vivo. Subsequent studies are essential to validate the observed synergistic effects and to ensure the safety and efficacy of the combined treatment approach before clinical trials in humans can be considered.

## 4. Materials and Methods

### 4.1. Cell Culture

The human chondrosarcoma cell lines CAL-78 and SW1353 were propagated under standard cell culture conditions (37 °C and 5% CO_2_). RPMI 1640 media supplemented with 1% penicillin/streptomycin (P/S) and 20% fetal calf serum (FCS) was used for the CAL-78 cells. Dulbecco’s modified Eagle’s medium (DMEM)/F12 containing stable glutamine, 1.2 g/L NaHCO_3_, 10% FCS, and 1% P/S was used for the SW1353 cells (all reagents from PAN Biotech, Aidenbach, Germany).

### 4.2. Proliferation Assay after CPP Exposure

For the cold physical plasma (CPP) treatment, the kINPen MED (neoplas MED, Greifswald, Germany) was used. The gas flow of the carrier gas argon (Alphagaz 1; Air Liquide, Düsseldorf, Germany) was adjusted to 3.5 standard liters per minute. Moreover, 2 × 10^4^ cells were diluted in 200 µL medium and transferred to the wells of a 24 well plate and treated with CPP for 5 s, 10 s, and 20 s. The control treatment with carrier gas argon was also performed. After the treatment, 800 μL fully supplemented cell culture medium was added to each well. The CASY cell counter and analyzer model TT (Roche Applied Science, Mannheim, Germany) was used to determine the number of viable cells after 4 h, 24 h, 48 h, 72 h, 96 h, and 120 h.

### 4.3. Chemotherapeutics

The cytostatic drugs cisplatin (CIS), doxorubicin (DOX), and vincristine (VIN) were used for the treatment of the chondrosarcoma cells. All the chemotherapeutics were obtained from Cayman Chemical (Ann Arbor, MI, USA). The cytostatic drugs were dissolved in DMSO (Carl Roth, Karlsruhe, Germany) to prepare the stock solutions. CIS was prepared at concentrations of 10^−2^ M, DOX was prepared at 10^−2^ M, and VIN was prepared at concentrations of 10^−3^ M. The stock solutions were then diluted to the desired concentrations using a dilution series in DMSO.

### 4.4. Cell Viability Assay after Cytostatic Exposure

The CellTiter-Blue Cell Viability Assay (Promega, Walldorf, Germany) was utilized to determine the effects of the cytostatic agents on the human chondrosarcoma lines. The cells were kept under standard cell culture conditions for 24 h before being treated with various concentrations of the cytostatic drugs or DMSO as a control. The cytostatic drug preparations were performed by diluting the stock solution to the desired concentrations in DMSO. The solutions were further diluted 1:100 in full medium. The cells were treated with the cytostatic-containing, fully supplemented cell culture medium and incubated for 72 h under standard cell culture conditions. The CellTiter-Blue reagent was added to the media, and the fluorescence signal was measured with a multimode plate reader at 560Ex/590Em (TECAN, Männedorf, Switzerland) after 2 h of incubation. The fluorescence signals of the cells treated with the cytostatic drugs were then normalized to the signals of the cells treated with DMSO (control) to determine their respective cell viability relative to the control.

### 4.5. Cell Viability Assay after Cytostatic and CPP Exposure

Cells were seeded into 96-well plates and incubated for 24 h prior to the combination therapy. The CPP treatment was carried out indirectly by transferring CPP-activated cell culture medium (PAM). For this, 200 µL full medium was transferred into the wells of a 24-well plate, and each well was treated with 5 s, 10 s, or 20 s CPP. PAM was added to the cells in the 96-well plates, followed by the addition of 100 μL complete medium containing the cytostatic drugs or DMSO. The CellTiter-blue assay was performed as described in the previous paragraph.

### 4.6. Proliferation Assay after Cytostatic and CPP Exposure

To assess the effect of combining the cytostatic agents with CPP on the cell growth of human chondrosarcoma cells, the growth kinetics were performed over 120 h. For this, 5 × 10^4^ cells in 200 μL medium were treated with CPP for 5 s. After the CPP exposure, 800 μL of warm, fully supplemented cell culture medium containing the corresponding cytostatic agent was added to each well. The cytostatic agents were used at their determined IC_20_ values. The control cells were treated with only the cytostatic agents and the carrier gas argon. The cells were incubated for 120 h under standard cell culture conditions. The number of viable cells was determined after 4 h, 24 h, 28 h, 72 h, 96 h, and 120 h using the Invitrogen Countess II Automated Cell Counter. The cells were stained 1:1 with 0.4% Trypan Blue (Invitrogen, Thermo Fisher Scientific, Schwerte, Germany).

### 4.7. Detection of Caspase 3/7 Activity

The CellEvent Caspase 3/7 Green Detection Reagent (Thermo Fisher Scientific, Waltham, MA, USA) was used to detect apoptosis. A total of 5.0 × 10^4^ CAL-78 cells, as well as 2.0 × 10^4^ SW1353 cells, were seeded in a 96-well plate. The CPP treatment was performed indirectly by treating 200 μL of full medium with CPP or argon for 5 s in a 24-well plate. Then, 100 μL of the treated medium was transferred into the wells of the 96-well plate. After the CPP treatment, the cells were treated with full media containing the cytostatic drugs or DMSO (control). After the incubation period (24 h and 48 h), the medium was carefully aspirated, and 100 μL of caspase 3/7 detection solution was added to the wells of the 96-well plate and incubated for 45 min under standard cell culture conditions. The fluorescence was measured using the TECAN multimode plate reader at 495Ex/535Em. The fluorescence was normalized to the proportion of viable cells determined with the cytotoxicity assay.

### 4.8. TUNEL Assay

The treatment was performed similarly to the caspase assay. The TiterTACS assay containing TACS Sapphire (R&D Systems, Minneapolis, MN, USA) was performed 24 h and 48 h after treatment according to the manufacturer’s protocol. The absorption was normalized to the proportion of viable cells determined with the cytotoxicity assay.

### 4.9. Cytotoxicity Assay

The treatment was performed similarly to the caspase assay. The CytoTox Cytotoxicity Assay (Promega, Walldorf, Germany) was performed 24 h and 48 h after treatment according to the manufacturer’s protocol.

### 4.10. Cell Metabolism Assay

The treatment with CPP and the cytostatic drugs was performed similarly to the regime applied for the caspase assay. The CellTiter Glo 2.0 assay (Promega, Walldorf, Germany) was performed 48 h after treatment according to the manufacturer’s protocol. The chemiluminescent signal was normalized to the cellular nucleic acids, which were determined with CyQUANT assay (Thermo Fisher Scientific, Waltham, MA, USA) according to the manufacturer’s protocol. The chemiluminescent and fluorescence signals were measured using a multimode plate reader (TECAN, Männedorf, Switzerland). The relative cellular ATP content per cell of the cells treated with CPP, cytostatics, or combination therapy was normalized to the mean of the cells treated with argon and DMSO (control).

### 4.11. Glucose Consumption Assay

The treatment with CPP and the cytostatic drugs was performed similarly to the regime applied for the caspase assay. Samples were taken from the cell-free supernatant and frozen at −80 °C. The glucose concentration was determined after a 1:00 dilution using the Glucose-Glo Assay (Promega, Walldorf, Germany). The consumption was calculated from the difference in the glucose concentration after 48 h and the concentration at the beginning of the experiment. The consumption was normalized to the proportion of living cells, which was determined using the cytotoxicity assay.

### 4.12. Sequential Cytostatic and CPP Treatment

Here, 5 × 10^4^ CAL-78 cells and 2 × 10^4^ SW1353 cells were seeded into the wells of a 96-well plate. After 24 h of incubation, the medium was carefully aspirated and replaced with medium containing chemotherapeutics (IC_20_). After another 24 h, the chemotherapeutic-containing medium was aspirated, the cells were washed with DPBS, and CPP-treated medium (5 s treatment time) was added to the cells. In the other group, the treatment steps were performed in reverse order. After an additional 24 h of incubation, cell viability was determined using the CellTiter-Blue assay.

### 4.13. CPP Exposure of Cytostatics

The cytostatics CIS, DOX and VIN were suspended in cell culture medium. These cytostatics were then combined with medium that had been treated with CPP for 0 s, 5 s, 10 s, and 20 s. The cytostatics were exposed to the CPP-treated medium for 48 h. Subsequently, this medium was applied to SW1353 cells that had been seeded the previous day. The cell viability was determined using the CellTiter-Glo (Promega, Walldorf, Germany) assay after an additional 48 h of incubation. The solvent DMSO and the treatment with 0 s of CPP were used as controls. The viability of the treatments was normalized to the controls.

### 4.14. Data Analysis

For the data analysis and visualization, GraphPad Prism Version 9.5.1 (GraphPad Software, La Jolla, CA, USA) was used. The results of *p* ≤ 0.05 of at least three independent experiments were considered significant, and data were given as the mean ± SD. The differences were examined using a two-way ANOVA, followed by Bonferroni’s multiple comparison test. For the experiments with repeated measurements at different times (e.g., proliferation assay), repeated-measure two-way ANOVAs were used.

## Data Availability

Data can be shared upon request.

## References

[1] Limaiem F., Davis D.D., Sticco K.L. (2024). Chondrosarcoma. StatPearls.

[2] Thorkildsen J., Myklebust T.Å. (2023). The National Incidence of Chondrosarcoma of Bone; a Review. Acta Oncol..

[3] Cosci I., Del Fiore P., Mocellin S., Ferlin A. (2024). Gender Differences in Soft Tissue and Bone Sarcoma: A Narrative Review. Cancers.

[4] Weinschenk R.C., Wang W.-L., Lewis V.O. (2021). Chondrosarcoma. JAAOS—J. Am. Acad. Orthop. Surg..

[5] Damron T.A., Ward W.G., Stewart A. (2007). Osteosarcoma, Chondrosarcoma, and Ewing’s Sarcoma: National Cancer Data Base Report. Clin. Orthop. Relat. Res.®.

[6] Hwang S., Hameed M., Kransdorf M. (2023). The 2020 World Health Organization Classification of Bone Tumors: What Radiologists Should Know. Skelet. Radiol..

[7] van Praag (Veroniek) V.M., Rueten-Budde A.J., Ho V., Dijkstra P.D.S., van der Geest I.C., Bramer J.A., Schaap G.R., Jutte P.C., Schreuder H.B., Ploegmakers J.J.W. (2018). Incidence, Outcomes and Prognostic Factors during 25 Years of Treatment of Chondrosarcomas. Surg. Oncol..

[8] Evans H.L., Ayala A.G., Romsdahl M.M. (1977). Prognostic Factors in Chondrosarcoma of Bone. A Clinicopathologic Analysis with Emphasis on Histologic Grading. Cancer.

[9] Kinoshita H., Kamoda H., Hagiwara Y., Kinoshita S., Ohtori S., Yonemoto T. (2022). Prognostic Factors for Survival in Patients with High-Grade Chondrosarcoma. Cancer Diagn. Progn..

[10] Fiorenza F., Abudu A., Grimer R.J., Carter S.R., Tillman R.M., Ayoub K., Mangham D.C., Davies A.M. (2002). Risk Factors for Survival and Local Control in Chondrosarcoma of Bone. J. Bone Jt. Surgery. Br. Vol..

[11] Stevenson J.D., Laitinen M.K., Parry M.C., Sumathi V., Grimer R.J., Jeys L.M. (2018). The Role of Surgical Margins in Chondrosarcoma. Eur. J. Surg. Oncol..

[12] Zając A.E., Kopeć S., Szostakowski B., Spałek M.J., Fiedorowicz M., Bylina E., Filipowicz P., Szumera-Ciećkiewicz A., Tysarowski A., Czarnecka A.M. (2021). Chondrosarcoma-from Molecular Pathology to Novel Therapies. Cancers.

[13] Mery B., Espenel S., Guy J.-B., Rancoule C., Vallard A., Aloy M.-T., Rodriguez-Lafrasse C., Magné N. (2018). Biological Aspects of Chondrosarcoma: Leaps and Hurdles. Crit. Rev. Oncol./Hematol..

[14] Moussavi-Harami F., Mollano A., Martin J.A., Ayoob A., Domann F.E., Gitelis S., Buckwalter J.A. (2006). Intrinsic Radiation Resistance in Human Chondrosarcoma Cells. Biochem. Biophys. Res. Commun..

[15] Terek R.M., Schwartz G.K., Devaney K., Glantz L., Mak S., Healey J.H., Albino A.P. (1998). Chemotherapy and P-glycoprotein Expression in Chondrosarcoma. J. Orthop. Res..

[16] Kim D.W., Kim K.-O., Shin M.J., Ha J.H., Seo S.W., Yang J., Lee F.Y. (2009). siRNA-Based Targeting of Antiapoptotic Genes Can Reverse Chemoresistance in P-Glycoprotein Expressing Chondrosarcoma Cells. Mol. Cancer.

[17] Laroussi M. (2020). Cold Plasma in Medicine and Healthcare: The New Frontier in Low Temperature Plasma Applications. Front. Phys..

[18] Yan D., Talbot A., Nourmohammadi N., Cheng X., Canady J., Sherman J., Keidar M. (2015). Principles of Using Cold Atmospheric Plasma Stimulated Media for Cancer Treatment. Sci. Rep..

[19] Ji H.W., Kim H., Kim H.W., Yun S.H., Park J.E., Choi E.H., Kim S.J. (2020). Genome-Wide Comparison of the Target Genes of the Reactive Oxygen Species and Non-Reactive Oxygen Species Constituents of Cold Atmospheric Plasma in Cancer Cells. Cancers.

[20] Afrasiabi M., Tahmasebi G., Eslami E., Seydi E., Pourahmad J. (2022). Cold Atmospheric Plasma versus Cisplatin against Oral Squamous Cell Carcinoma: A Mitochondrial Targeting Study. Iran. J. Pharm. Res..

[21] Silva-Teixeira R., Laranjo M., Lopes B., Almeida-Ferreira C., Gonçalves A.C., Rodrigues T., Matafome P., Sarmento-Ribeiro A.B., Caramelo F., Botelho M.F. (2021). Plasma Activated Media and Direct Exposition Can Selectively Ablate Retinoblastoma Cells. Free Radic. Biol. Med..

[22] Sato Y., Yamada S., Takeda S., Hattori N., Nakamura K., Tanaka H., Mizuno M., Hori M., Kodera Y. (2018). Effect of Plasma-Activated Lactated Ringer’s Solution on Pancreatic Cancer Cells In Vitro and In Vivo. Ann. Surg. Oncol..

[23] Nitsch A., Stope M.B. (2021). The Future Therapy of Renal Cell Carcinoma? Non-Invasive Physical Plasma as an Innovative Oncological Therapy Modality. J. Cancer Ther..

[24] Dai X., Wu J., Lu L., Chen Y. (2023). Current Status and Future Trends of Cold Atmospheric Plasma as an Oncotherapy. Biomol. Ther..

[25] Nitsch A., Strakeljahn S., Jacoby J.M., Sieb K.F., Mustea A., Bekeschus S., Ekkernkamp A., Stope M.B., Haralambiev L. (2022). New Approach against Chondrosoma Cells—Cold Plasma Treatment Inhibits Cell Motility and Metabolism, and Leads to Apoptosis. Biomedicines.

[26] Haralambiev L., Nitsch A., Jacoby J.M., Strakeljahn S., Bekeschus S., Mustea A., Ekkernkamp A., Stope M.B. (2020). Cold Atmospheric Plasma Treatment of Chondrosarcoma Cells Affects Proliferation and Cell Membrane Permeability. Int. J. Mol. Sci..

[27] Murillo D., Huergo C., Gallego B., Rodríguez R., Tornín J. (2023). Exploring the Use of Cold Atmospheric Plasma to Overcome Drug Resistance in Cancer. Biomedicines.

[28] Mateu-Sanz M., Ginebra M.-P., Tornín J., Canal C. (2022). Cold Atmospheric Plasma Enhances Doxorubicin Selectivity in Metastasic Bone Cancer. Free Radic. Biol. Med..

[29] Pefani-Antimisiari K., Athanasopoulos D.K., Marazioti A., Sklias K., Rodi M., de Lastic A.-L., Mouzaki A., Svarnas P., Antimisiaris S.G. (2021). Synergistic Effect of Cold Atmospheric Pressure Plasma and Free or Liposomal Doxorubicin on Melanoma Cells. Sci. Rep..

[30] Lee S., Lee H., Jeong D., Ham J., Park S., Choi E.H., Kim S.J. (2017). Cold Atmospheric Plasma Restores Tamoxifen Sensitivity in Resistant MCF-7 Breast Cancer Cell. Free Radic. Biol. Med..

[31] Yan D., Sherman J.H., Keidar M. (2016). Cold Atmospheric Plasma, a Novel Promising Anti-Cancer Treatment Modality. Oncotarget.

[32] Yan D., Wang Q., Malyavko A., Zolotukhin D.B., Adhikari M., Sherman J.H., Keidar M. (2020). The Anti-Glioblastoma Effect of Cold Atmospheric Plasma Treatment: Physical Pathway vs. Chemical Pathway. Sci. Rep..

[33] Bekeschus S., Moritz J., Helfrich I., Boeckmann L., Weltmann K.-D., Emmert S., Metelmann H.-R., Stoffels I., von Woedtke T. (2020). Ex Vivo Exposure of Human Melanoma Tissue to Cold Physical Plasma Elicits Apoptosis and Modulates Inflammation. Appl. Sci..

[34] Almeida-Ferreira C., Silva-Teixeira R., Gonçalves A.C., Marto C.M., Sarmento-Ribeiro A.B., Caramelo F., Botelho M.F., Laranjo M. (2022). Cold Atmospheric Plasma Apoptotic and Oxidative Effects on MCF7 and HCC1806 Human Breast Cancer Cells. Int. J. Mol. Sci..

[35] Cheng X., Rowe W., Ly L., Shashurin A., Zhuang T., Wigh S., Basadonna G., Trink B., Keidar M., Canady J. (2018). Treatment of Triple-Negative Breast Cancer Cells with the Canady Cold Plasma Conversion System: Preliminary Results. Plasma.

[36] Nitsch A., Sieb K.F., Qarqash S., Schoon J., Ekkernkamp A., Wassilew G.I., Niethard M., Haralambiev L. (2023). Selective Effects of Cold Atmospheric Plasma on Bone Sarcoma Cells and Human Osteoblasts. Biomedicines.

[37] Haralambiev L., Nitsch A., Einenkel R., Muzzio D.O., Gelbrich N., Burchardt M., Zygmunt M., Ekkernkamp A., Stope M.B., Gümbel D. (2020). The Effect of Cold Atmospheric Plasma on the Membrane Permeability of Human Osteosarcoma Cells. Anticancer Res..

[38] Semmler M.L., Bekeschus S., Schäfer M., Bernhardt T., Fischer T., Witzke K., Seebauer C., Rebl H., Grambow E., Vollmar B. (2020). Molecular Mechanisms of the Efficacy of Cold Atmospheric Pressure Plasma (CAP) in Cancer Treatment. Cancers.

[39] Köritzer J., Boxhammer V., Schäfer A., Shimizu T., Klämpfl T.G., Li Y.-F., Welz C., Schwenk-Zieger S., Morfill G.E., Zimmermann J.L. (2013). Restoration of Sensitivity in Chemo—Resistant Glioma Cells by Cold Atmospheric Plasma. PLoS ONE.

[40] Gjika E., Pal-Ghosh S., Kirschner M.E., Lin L., Sherman J.H., Stepp M.A., Keidar M. (2020). Combination Therapy of Cold Atmospheric Plasma (CAP) with Temozolomide in the Treatment of U87MG Glioblastoma Cells. Sci. Rep..

[41] Yao X., Yan D., Lin L., Sherman J.H., Peters K.B., Keir S.T., Keidar M. (2022). Cold Plasma Discharge Tube Enhances Antitumoral Efficacy of Temozolomide. ACS Appl. Bio Mater..

[42] Sagwal S.K., Pasqual-Melo G., Bodnar Y., Gandhirajan R.K., Bekeschus S. (2018). Combination of Chemotherapy and Physical Plasma Elicits Melanoma Cell Death via Upregulation of SLC22A16. Cell Death Dis..

[43] Alimohammadi M., Golpour M., Sohbatzadeh F., Hadavi S., Bekeschus S., Niaki H.A., Valadan R., Rafiei A. (2020). Cold Atmospheric Plasma Is a Potent Tool to Improve Chemotherapy in Melanoma In Vitro and In Vivo. Biomolecules.

[44] Dezhpour A., Ghafouri H., Jafari S., Nilkar M. (2023). Effects of Cold Atmospheric-Pressure Plasma in Combination with Doxorubicin Drug against Breast Cancer Cells in Vitro and in Vivo. Free Radic. Biol. Med..

[45] Zahedian S., Hekmat A., Tackallou S.H., Ghoranneviss M. (2022). The Impacts of Prepared Plasma-Activated Medium (PAM) Combined with Doxorubicin on the Viability of MCF-7 Breast Cancer Cells: A New Cancer Treatment Strategy. Rep. Biochem. Mol. Biol..

[46] Park S., Kim H., Ji H.W., Kim H.W., Yun S.H., Choi E.H., Kim S.J. (2019). Cold Atmospheric Plasma Restores Paclitaxel Sensitivity to Paclitaxel-Resistant Breast Cancer Cells by Reversing Expression of Resistance-Related Genes. Cancers.

[47] Liedtke K.R., Diedrich S., Pati O., Freund E., Flieger R., Heidecke C.D., Partecke L.I., Bekeschus S. (2018). Cold Physical Plasma Selectively Elicits Apoptosis in Murine Pancreatic Cancer Cells *In Vitro* and *In Ovo*. Anticancer Res..

[48] Masur K., von Behr M., Bekeschus S., Weltmann K.-D., Hackbarth C., Heidecke C.-D., von Bernstorff W., von Woedtke T., Partecke L.I. (2015). Synergistic Inhibition of Tumor Cell Proliferation by Cold Plasma and Gemcitabine. Plasma Process. Polym..

[49] Nitsch A., Qarqash S., Römer S., Schoon J., Ekkernkamp A., Niethard M., Reichert J.C., Wassilew G.I., Tzvetkov M.V., Haralambiev L. (2023). Enhancing the Impact of Chemotherapy on Ewing Sarcoma Cells through Combination with Cold Physical Plasma. Int. J. Mol. Sci..

[50] Kim C.-H., Bahn J.H., Lee S.-H., Kim G.-Y., Jun S.-I., Lee K., Baek S.J. (2010). Induction of Cell Growth Arrest by Atmospheric Non-Thermal Plasma in Colorectal Cancer Cells. J. Biotechnol..

[51] Koensgen D., Besic I., Guembel D., Kaul A., Weiss M., Diesing K., Kramer A., Bekeschus S., Mustea A., Stope M.B. (2017). Cold Atmospheric Plasma (CAP) and CAP-Stimulated Cell Culture Media Suppress Ovarian Cancer Cell Growth—A Putative Treatment Option in Ovarian Cancer Therapy. Anticancer Res..

[52] Gazendam A., Popovic S., Parasu N., Ghert M. (2023). Chondrosarcoma: A Clinical Review. J. Clin. Med..

[53] Bovée J.V., Cleton-Jansen A.-M., Taminiau A.H., Hogendoorn P.C. (2005). Emerging Pathways in the Development of Chondrosarcoma of Bone and Implications for Targeted Treatment. Lancet Oncol..

[54] Italiano A., Mir O., Cioffi A., Palmerini E., Piperno-Neumann S., Perrin C., Chaigneau L., Penel N., Duffaud F., Kurtz J.E. (2013). Advanced Chondrosarcomas: Role of Chemotherapy and Survival. Ann. Oncol..

[55] van Oosterwijk J.G., Herpers B., Meijer D., Briaire-de Bruijn I.H., Cleton-Jansen A.M., Gelderblom H., van de Water B., Bovée J.V.M.G. (2012). Restoration of Chemosensitivity for Doxorubicin and Cisplatin in Chondrosarcoma in Vitro: BCL-2 Family Members Cause Chemoresistance. Ann. Oncol..

[56] Tian Z., Yao W. (2023). Chemotherapeutic Drugs for Soft Tissue Sarcomas: A Review. Front. Pharmacol..

[57] Cairns R.A., Harris I.S., Mak T.W. (2011). Regulation of Cancer Cell Metabolism. Nat. Rev. Cancer.

[58] Apicella M., Giannoni E., Fiore S., Ferrari K.J., Fernández-Pérez D., Isella C., Granchi C., Minutolo F., Sottile A., Comoglio P.M. (2018). Increased Lactate Secretion by Cancer Cells Sustains Non-Cell-Autonomous Adaptive Resistance to MET and EGFR Targeted Therapies. Cell Metab..

[59] Guo B., Pomicter A.D., Li F., Bhatt S., Chen C., Li W., Qi M., Huang C., Deininger M.W., Kong M.G. (2021). Trident Cold Atmospheric Plasma Blocks Three Cancer Survival Pathways to Overcome Therapy Resistance. Proc. Natl. Acad. Sci. USA.

[60] Mokhtari H., Farahmand L., Yaserian K., Jalili N., Majidzadeh-A K. (2019). The Antiproliferative Effects of Cold Atmospheric Plasma-Activated Media on Different Cancer Cell Lines, the Implication of Ozone as a Possible Underlying Mechanism. J. Cell. Physiol..

[61] Wende K., von Woedtke T., Weltmann K.-D., Bekeschus S. (2019). Chemistry and Biochemistry of Cold Physical Plasma Derived Reactive Species in Liquids. Biol. Chem..

[62] Wenske S., Lackmann J.-W., Busch L.M., Bekeschus S., von Woedtke T., Wende K. (2021). Reactive Species Driven Oxidative Modifications of Peptides—Tracing Physical Plasma Liquid Chemistry. J. Appl. Phys..

[63] Bauer G., Graves D.B. (2016). Mechanisms of Selective Antitumor Action of Cold Atmospheric Plasma-Derived Reactive Oxygen and Nitrogen Species. Plasma Process. Polym..

[64] Yan X., Zou F., Zhao S., Lu X., He G., Xiong Z., Xiong Q., Zhao Q., Deng P., Huang J. (2010). On the Mechanism of Plasma Inducing Cell Apoptosis. IEEE Trans. Plasma Sci..

[65] Thiyagarajan M., Anderson H., Gonzales X.F. (2014). Induction of Apoptosis in Human Myeloid Leukemia Cells by Remote Exposure of Resistive Barrier Cold Plasma. Biotech. Bioeng..

[66] Yazdani Z., Pasandi M.S., Golpour M., Eslami M., Rafiei A. (2024). Effect of Cold Atmospheric Plasma on Changing of Biomolecular Structures Involved in Apoptosis Pathways of Melanoma Cancer. Ski. Res. Technol..

[67] Sersenová D., Machala Z., Repiská V., Gbelcová H. (2021). Selective Apoptotic Effect of Plasma Activated Liquids on Human Cancer Cell Lines. Molecules.

[68] Lee C.-M., Jeong Y.-I., Kook M.-S., Kim B.-H. (2020). Combinatorial Effect of Cold Atmosphere Plasma (CAP) and the Anticancer Drug Cisplatin on Oral Squamous Cell Cancer Therapy. Int. J. Mol. Sci..

[69] Wende K., Bekeschus S., Schmidt A., Jatsch L., Hasse S., Weltmann K.D., Masur K., von Woedtke T. (2016). Risk Assessment of a Cold Argon Plasma Jet in Respect to Its Mutagenicity. Mutat. Res./Genet. Toxicol. Environ. Mutagen..

[70] Kluge S., Bekeschus S., Bender C., Benkhai H., Sckell A., Below H., Stope M.B., Kramer A. (2016). Investigating the Mutagenicity of a Cold Argon-Plasma Jet in an HET-MN Model. PLoS ONE.

[71] Jablonowski L., Kocher T., Schindler A., Müller K., Dombrowski F., von Woedtke T., Arnold T., Lehmann A., Rupf S., Evert M. (2019). Side Effects by Oral Application of Atmospheric Pressure Plasma on the Mucosa in Mice. PLoS ONE.

[72] Rutkowski R., Daeschlein G., von Woedtke T., Smeets R., Gosau M., Metelmann H.-R. (2020). Long-Term Risk Assessment for Medical Application of Cold Atmospheric Pressure Plasma. Diagnostics.

[73] Schuster M., Rutkowski R., Hauschild A., Shojaei R.K., von Woedtke T., Rana A., Bauer G., Metelmann P., Seebauer C. (2018). Side Effects in Cold Plasma Treatment of Advanced Oral Cancer—Clinical Data and Biological Interpretation. Clin. Plasma Med..

